# Crescent shaped Fabry-Perot fiber cavity for ultra-sensitive strain measurement

**DOI:** 10.1038/srep38390

**Published:** 2016-12-02

**Authors:** Ye Liu, D. N. Wang, W. P. Chen

**Affiliations:** 1 College of Optical and Electronic Technology China Jiliang University, Hangzhou, 310018, China

## Abstract

Optical Fabry-Perot interferometer sensors based on inner air-cavity is featured with compact size, good robustness and high strain sensitivity, especially when an ultra-thin air-cavity is adopted. The typical shape of Fabry-Perot inner air-cavity with reflection mode of operation is elliptic, with minor axis along with and major axis perpendicular to the fiber length. The first reflection surface is diverging whereas the second one is converging. To increase the visibility of the output interference pattern, the length of major axis should be large for a given cavity length. However, the largest value of the major axis is limited by the optical fiber diameter. If the major axis length reaches the fiber diameter, the robustness of the Fabry-Perot cavity device would be decreased. Here we demonstrate an ultra-thin crescent shaped Fabry-Perot cavity for strain sensing with ultra-high sensitivity and low temperature cross-sensitivity. The crescent-shape cavity consists of two converging reflection surfaces, which provide the advantages of enhanced strain sensitivity when compared with elliptic or D-shaped FP cavity. The device is fabricated by fusion splicing an etched multimode fiber with a single mode fiber, and hence is simple in structure and economic in cost.

Optical fiber strain sensors have been attractive in many industrial and engineering applications due to their features of compact size, flexible operation, resistant to corrosion, immunity to electromagnetic interference and suitability for monitoring harsh environment. Among various types of optical fiber strain sensors demonstrated, such as those based on fiber gratings^1^,^2^,^3^, photonics crystal fibers (PCFs)^4^,^5^,^6^,^7^ and tapered optical fibers or microfibers^8^,^9^,^10^, fiber in-line interferometers^11^,^12^,^13^,^14^ have been developed rapidly, owing to their high sensitivity, ease of construction and convenience in operation. The main configurations of optical fiber in-line interferometer strain sensors include Mach-Zehnder interferometer (MZI) and Fabry–Pérot interferometer (FPI), based on spheroidal cavity^14^, hollow tube or PCF^5^,^15^,^16^,^17^, open air-cavity^18^, fiber taper or microfiber^9^,^10^,^19^ or lateral-shifted fiber splicing^12^ respectively. These sensors have large size, complex structure, poor robustness, and/or low strain sensitivity.

An elegant way of constructing a compact size, simple structure, good robustness and high sensitivity optical fiber in-line interferometer strain sensor is to utilize a fiber inner air-cavity, especially that with small cavity length.

Here, we propose and demonstrate a crescent shaped fiber FP cavity fabricated by fusion splicing an etched multimode fiber (MMF) with a single mode fiber (SMF) for strain sensing. Such an FP cavity device is highly compact, robust, low cost, simple in structure and easy in fabrication, and exhibits ultra-high strain sensitivity.

## Operating principle

**Figure 1:**
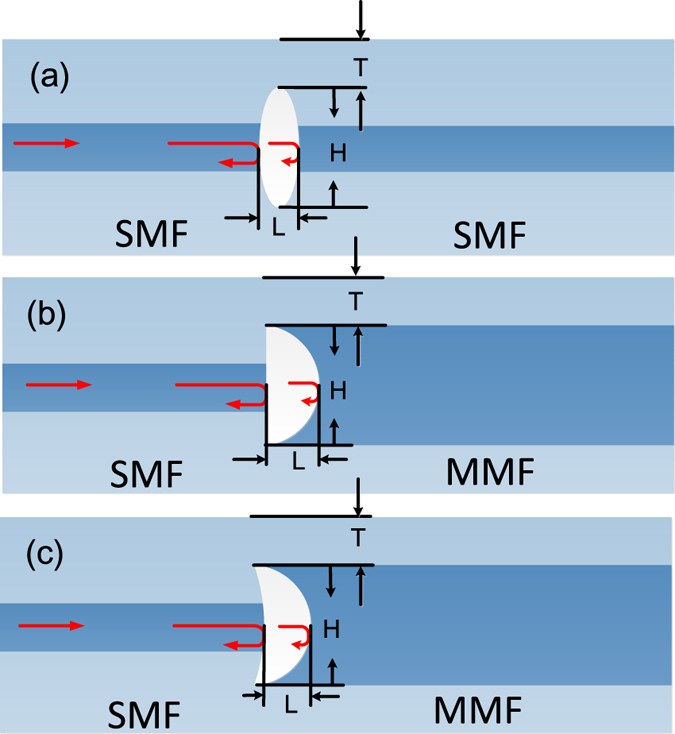
(**a**) Elliptic FP cavity; (**b**) D-shaped FP cavity; (**c**) Crescent shaped FP cavity.

The fiber devices with elliptic, D- and crescent shaped FP cavities respectively, are shown in Fig. 1, where L represents the cavity length, H denotes the height of the cavity and T is the thickness of the cavity wall.

In these FP cavities, the first reflection surfaces are diverging, flat and converging for the elliptic, D- and crescent shaped cavities respectively, while the second reflection surfaces are all converging.

The incident light beam traveling along the fiber core of SMF is reflected by the two surfaces of the FP cavity respectively and recombined in the fiber core, resulting in an interference fringe pattern at the output.

Assuming that the light intensities of the reflected beams by the two surfaces of the FP cavity are I_1_ and I_2_, respectively, the interference signal intensity can be written as:


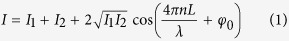


where λ is the wavelength of the incident light, n is the refractive index (RI) of the cavity medium, L is cavity length and φ_0_ is the initial phase of the interference. At the output fringe dip positions, the phase difference of the two reflected light beams satisfies the condition,


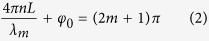


where m is an integer, λ_m_ is the wavelength of the m^th^ order interference dip. Assuming that φ_0_ = 0, when the condition 
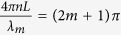
 is satisfied, the intensity dip appears at the wavelength


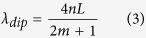


The free spectral range in the spectrum can then be expressed as,


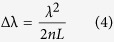


For an air cavity, n = 1, the dip wavelength shift due to axial strain can be derived from Eq. (3) as


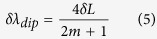


where *δL* is the change of air cavity length.

## Experimental Results and Discussion

**Figure 2:**
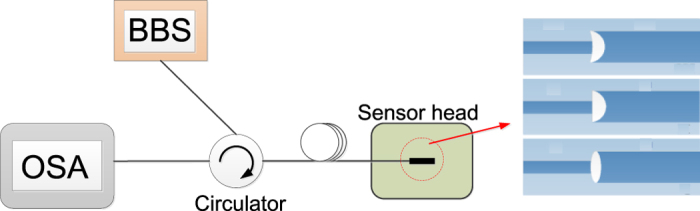
Schematic diagram of the experimental set-up.

The simple experimental set-up is demonstrated in Fig. 2. The incident light beam from a broadband (BBS) light source with wavelength range between 1452 to 1652 nm is launched into the fiber device via a circulator and the output is directed to an optical spectrum analyzer (OSA) (YOKOGAWA 6390) with the resolution of 0.01 nm to record its spectrum.

**Figure 3:**
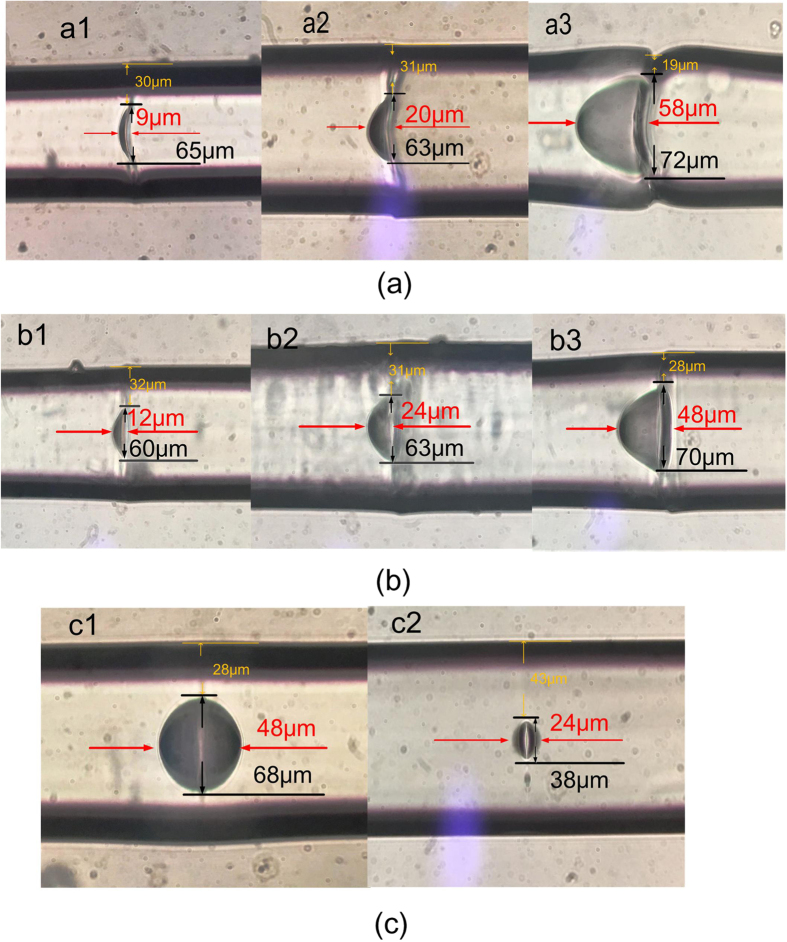
The microscope images of device samples. (**a**) Three device samples with crescent shaped FP cavity. (**b**) Three device samples with D-shaped FP cavity. (**c**) Two device samples with elliptic FP cavity.

The microscope images of FP cavity device samples with different shapes and sizes are displayed in Fig. 3, where crescent, D-shaped and elliptic FP cavities can be clearly observed. The cavity length, height and wall thickness of the device samples are summarized in Table 1.

During the experiment implementation, the fiber device was mounted between a fixed stage and a moving stage, and the instant adhesive was used to fix the fiber. The axial strain was applied by adjusting the translation stage to introduce a displacement in the whole fiber device length, and the range of strain was between 0 and 3000 με.

**Figure 4:**
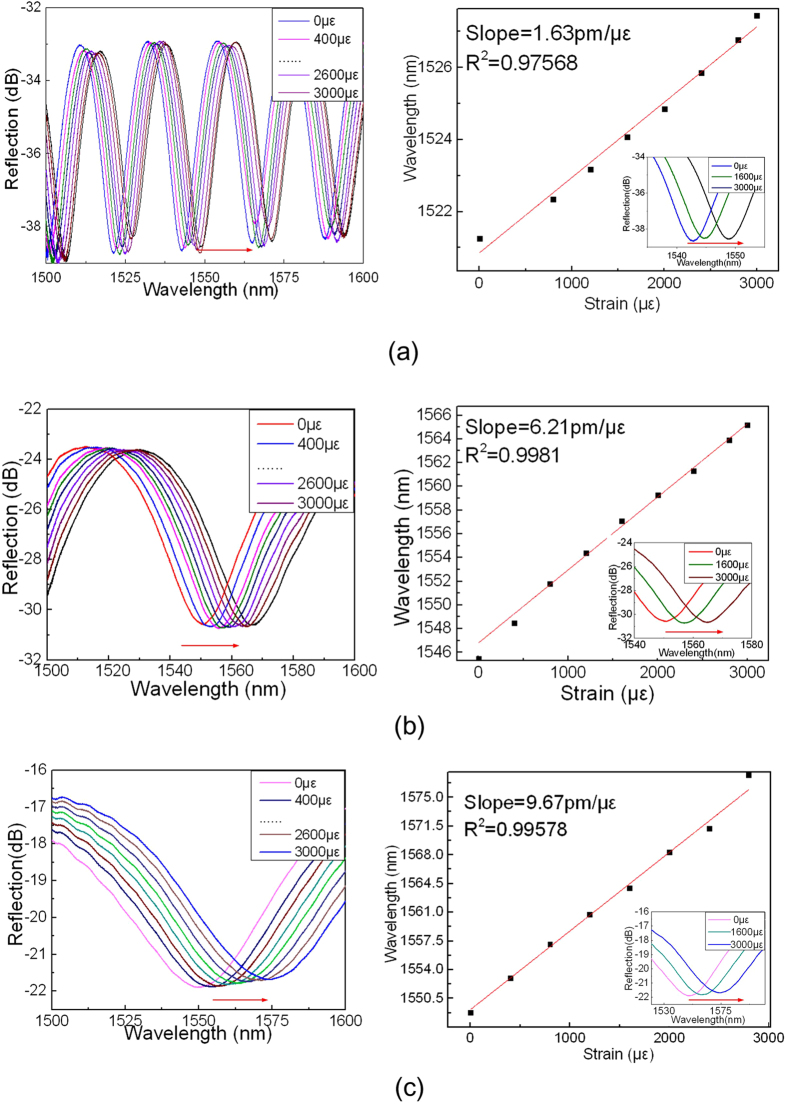
The reflection spectra and the output dip wavelength versus axial strain for device sample of crescent shaped FP cavity with (**a**) cavity length of 58 μm; (**b**) cavity length of 20 μm; and (**c**) cavity length of 9 μm.

The reflection spectra and the output dip wavelength versus axial strain for three device samples of crescent shaped FP cavities with cavity lengths of 58, 20 and 9 μm, respectively, are displayed in Fig. 4. The dip wavelength shift exhibits a good linear relationship with axial strain applied and the sensitivities achieved are 1.63, 6.21 and 9.67 pm/με, respectively.

**Figure 5:**
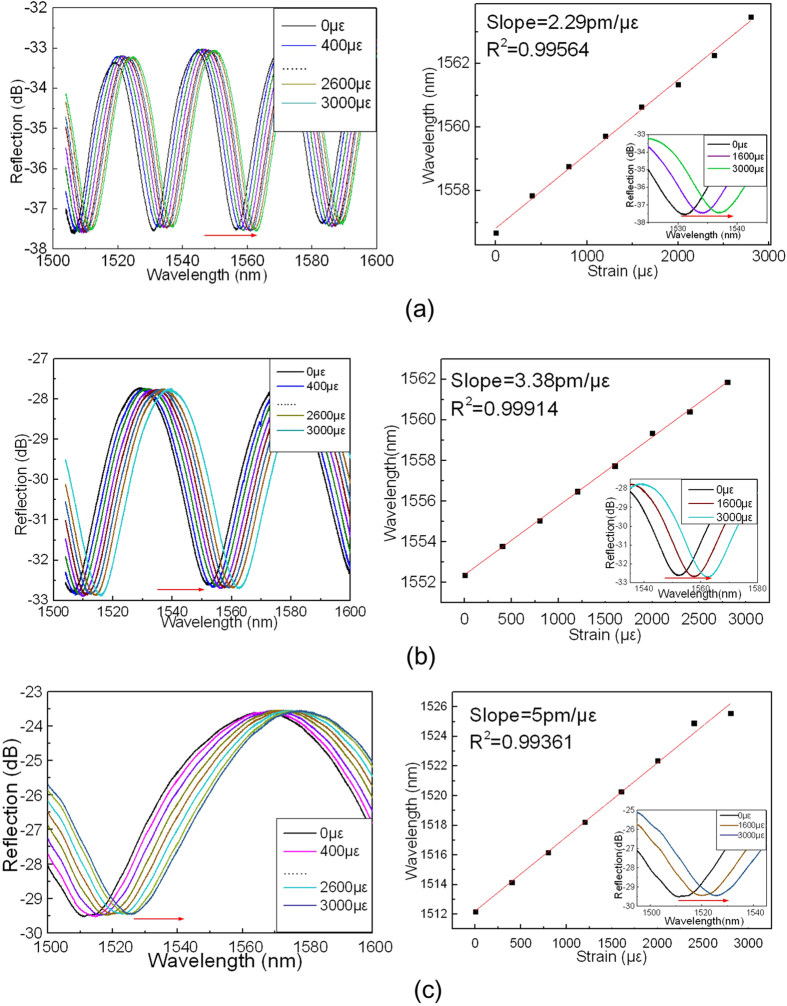
The reflection spectra and the output dip wavelength versus axial strain for device sample of D-shaped FP cavity with (**a**) cavity length of 48 μm; (**b**) cavity length of 24 μm; and (**c**) cavity length of 12 μm.

Figure 5 demonstrate the reflection spectra and the output dip wavelength shift versus axial strain for three device samples of D-shaped FP cavities with cavity lengths of 48, 24 and 12 μm, respectively. The dip wavelength shift exhibits a good linear relationship with the axial strain applied and the sensitivities obtained are 2.29, 3.38 and 5 pm/με, respectively.

**Figure 6:**
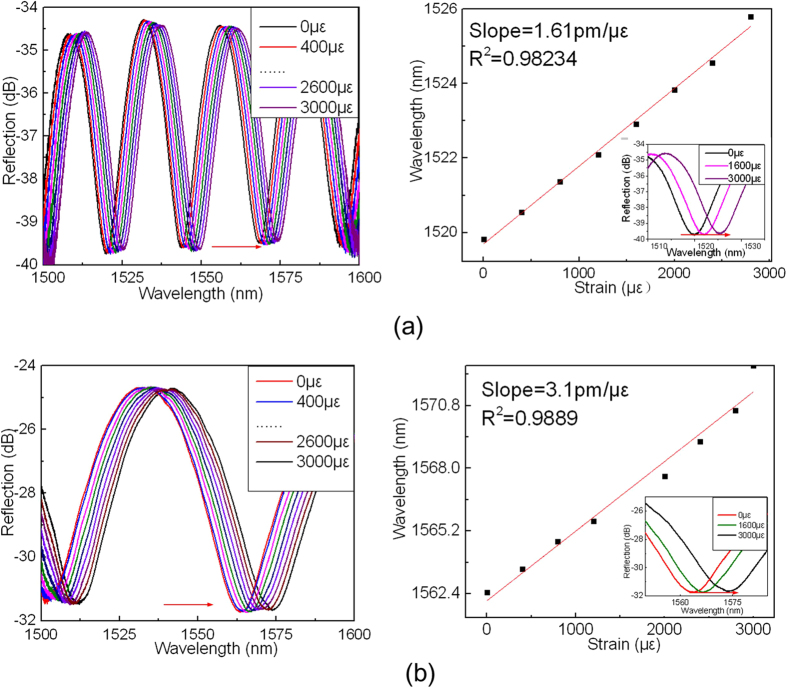
The reflection spectra and the output dip wavelength versus axial strain for device sample of elliptic FP cavity with (**a**) cavity length of 48 μm; (**b**) cavity length of 24 μm.

For comparison, the transmission spectra and the output dip wavelength shift versus axial strain for the device samples with elliptic FP cavity are shown in Fig. 6. Such samples are fabricated by fusion splicing a section of MMF etched by hydrofluoric (HF) acid with a section of SMF. In Fig. 6(a), the cavity length is ~48 μm, and the strain sensitivity obtained is 1.61 pm/με. In Fig. 6(b), the cavity length becomes ~24 μm, and the strain sensitivity obtained is ~3.1 pm/με.

It can be seen from Fig. 4 to Fig. 6 that the strain sensitivity depends on both the FP cavity length and the cavity shape. For the same cavity shape, the smaller the cavity length, the higher the strain sensitivity achieved. The D-shaped cavity exhibits the higher strain sensitivity than that of elliptic cavity, while the crescent shaped cavity possesses the highest strain sensitivity. This may be due to the fact that the crescent-shaped FP cavity has two converging reflection surfaces, compared with one flat and one converging, and one diverging and one converging reflection surfaces of D-shaped and elliptic shaped cavities, respectively. The converging, flat or diverging reflected beams not only affect the insertion loss of the device but also the variation of cavity length. The insertion loss of the crescent shaped FP cavity is also smaller than that of the other shaped cavities, and the smaller the cavity length, the lower the insertion loss. The smallest insertion loss achieved by crescent shaped cavity with cavity length of 9 μm is ~16 dB. The visibility of the crescent shaped FP cavity also appears to be the best of the three type FP cavities. For the device samples with cavity length of ~20 μm, the crescent shaped FP cavity has a visibility of 7 dB, compared with the results of 5 and 6.5 dB of the D-shaped and elliptic FP cavities with cavity length of ~24 μm. It can also be noted that the thickness of cavity wall plays no clearly significant role when compared with the cavity length, being different from that of the previous work reported^20^. This is likely due to the fact that our device samples exhibit only a short length of cavity wall (at least one reflection surface is a curve) associated with a relatively large thickness.

**Figure 7:**
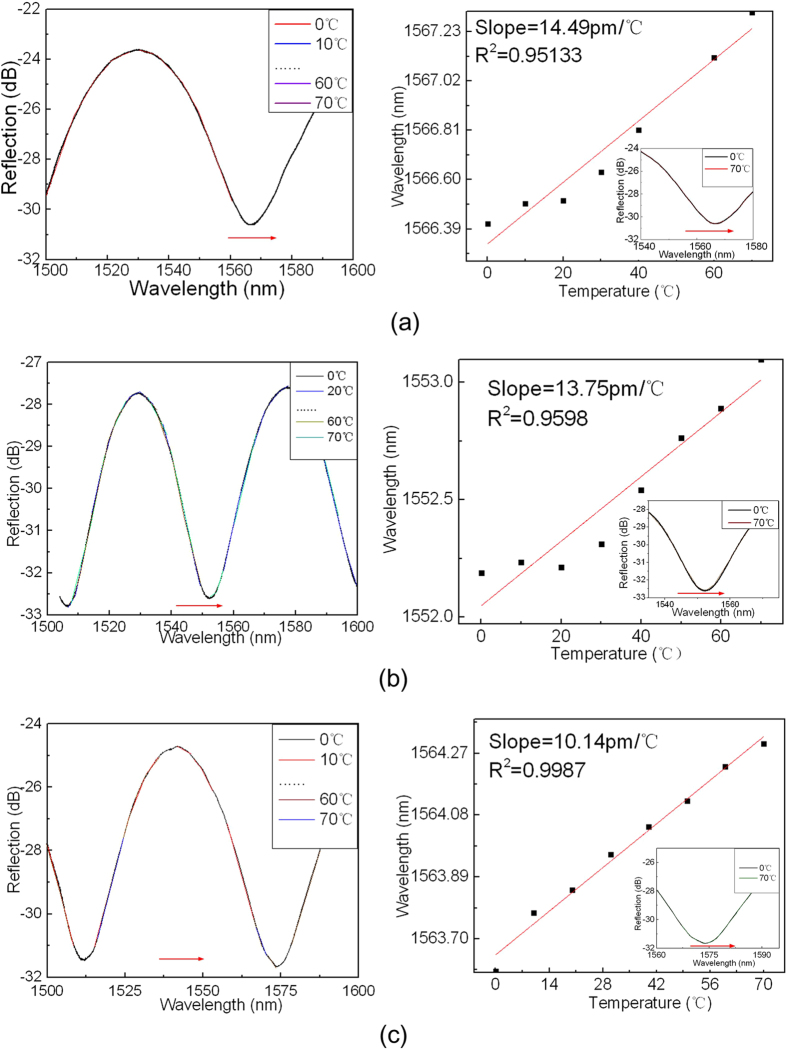
The reflection spectra and the output dip wavelength versus temperature for device sample of (**a**) crescent shaped FP cavity with cavity length of 20 μm; (**b**) D-shaped FP cavity with cavity length of 24 μm; (**c**) elliptic FP cavity with cavity length of 24 μm.

The temperature responses of the FP cavity devices of different shapes are displayed in Fig. 7. The temperature sensitivities for a crescent shaped FP cavity with cavity length of 20 μm, a D-shaped FP cavity with cavity length of 24 μm, and an elliptic FP cavity with cavity length of 24 μm, are ~15.45, ~12.41, and ~10.74 pm/°C, respectively. Considering of their strain sensitivity of ~6.21, ~3.38, and ~3.1 pm/με respectively, the temperature cross-sensitivities are determined as ~2.49, ~3.67 and ~3.46 με/°C respectively, which are lower than that of MZI (5.25 με/ °C)^21^ and PCF based Sagnac loop^22^. The crescent shaped FP cavity also exhibits the lowest temperature cross-sensitivity when compared with that of D-shaped and elliptic FP cavities.

## Conclusion

In conclusion we have demonstrated an ultra-thin crescent shaped FP cavity for ultra-sensitive strain measurement. The device is simply fabricated by fusion splicing an etched MMF with a section of SMF. The smallest cavity length achieved is ~9 μm, which provides a high sensitivity of ~9.67 pm/μm, more than 3 times that of optical fiber MZI and ~9 times that of fiber Bragg grating. The crescent shaped FP cavity is superior to other FP cavities such as elliptic and D-shaped cavities in terms of strain sensitivity, insertion loss, fringe visibility as well as temperature cross-sensitivity. Such an FP cavity device is highly compact, robust, low cost, simple in structure and easy in fabrication, and exhibits ultra-high strain sensitivity and low temperature cross-sensitivity, thus is promising in many strain sensing applications.

## Methods

### Device fabrication

**Figure 8:**
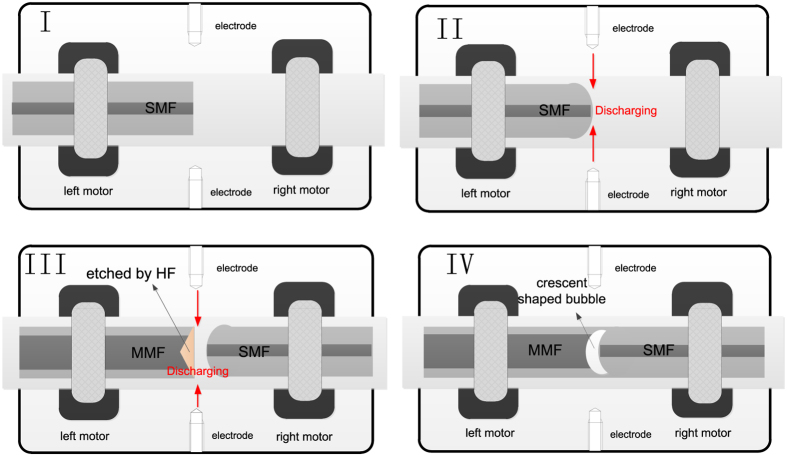
Schematic diagram of the crescent shaped FP cavity fabrication process.

The crescent shaped FP cavity device is fabricated by fusion splicing an etched MMF with a section of SMF. The fabrication process includes a number of steps which are illustrated in Fig. 8.

- The cleaved end of SMF is placed in a fusion splicer and electrically discharged for a short period of time to create an arching shaped fiber end.
- A section of MMF is etched by use of HF acid with concentration of 40% before a taper-shaped hole of several micrometers in depth is formed at the end of MMF.
- The etched MMF is then fusion spliced with the SMF with the arching shaped fiber end to form a crescent shaped inner air FP cavity.

During the device fabrication process of crescent shaped FP cavities, the three device samples with cavity lengths of 58, 20 and 9 μm, respectively, are etched for 3.5, 1.5 and 1 minutes, respectively, the discharge time and power employed in the fusion splicer (Fujikura 80 s) are 300 ms and 45 bit respectively, and the overlap adopted are 20, 15 and 12 μm, respectively.

**Figure 9:**
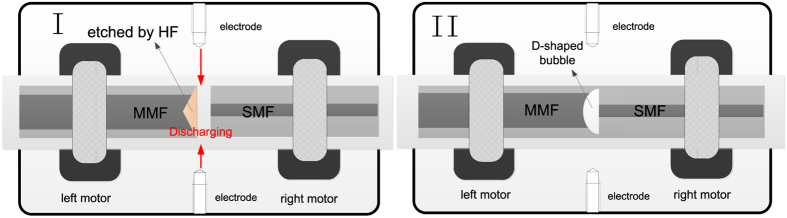
Schematic diagram of the D-shaped FP cavity fabrication process.

To create a D-shaped inner air FP cavity, the SMF with a cleaved fiber end is directly fusion spliced with etched MMF as demonstrated in Fig. 9. The etched time for three device samples with D-shaped FP cavity lengths of 48, 24 and 12 μm, respectively, are 3.5, 2 and 1 minute respectively, the discharge time and power employed are 300 ms and 40 bit respectively, and the overlap lengths adopted for all the three device samples are 10 μm.

**Figure 10:**
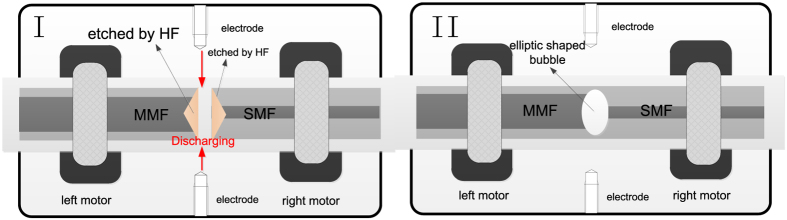
Schematic diagram of the elliptic FP cavity fabrication process.

The elliptic FP cavity is fabricated by fusion splicing of etched MMF and SMF as shown in Fig. 10. The etched time for the two device samples of elliptic FP cavity with cavity lengths of 48, and 24 μm, respectively are 5 and 2.5 minutes, respectively, the discharge time and power employed are 1000 ms and 30 bit respectively, and the overlap lengths adopted for both the device samples are 10 μm.

## Additional Information

**How to cite this article**: Liu, Y. *et al*. Crescent shaped Fabry-Perot fiber cavity for ultra-sensitive strain measurement. *Sci. Rep.* **6**, 38390; doi: 10.1038/srep38390 (2016).

**Publisher's note:** Springer Nature remains neutral with regard to jurisdictional claims in published maps and institutional affiliations.

## Figures and Tables

**Table 1:** FP cavity sample parameters.

Device sample	Cavity shape	Length (μm)	Height (μm)	Wall thickness (μm)
1	Crescent	9	65	48
2	Crescent	20	63	47
3	Crescent	58	72	17
4	D-shaped	12	60	43
5	D-shaped	24	63	48
6	D-shaped	48	70	26
7	Elliptic	48	68	38
8	Elliptic	24	38	44
